# Supplementary material for: Venomix: a simple bioinformatic pipeline for identifying and characterizing toxin gene candidates from transcriptomic data
Source: PeerJ. 2018 Jul 31;6:e5361. doi: 10.7717/peerj.5361 (PMC6074769; doi:10.7717/peerj.5361)
Supplement: Supplemental Information 3 [file peerj-06-5361-s003.gz › FinalOutput_E-6/Toxins/Snake_venom_metalloproteinase_acutolysin-C_1/finaltree.pdf]

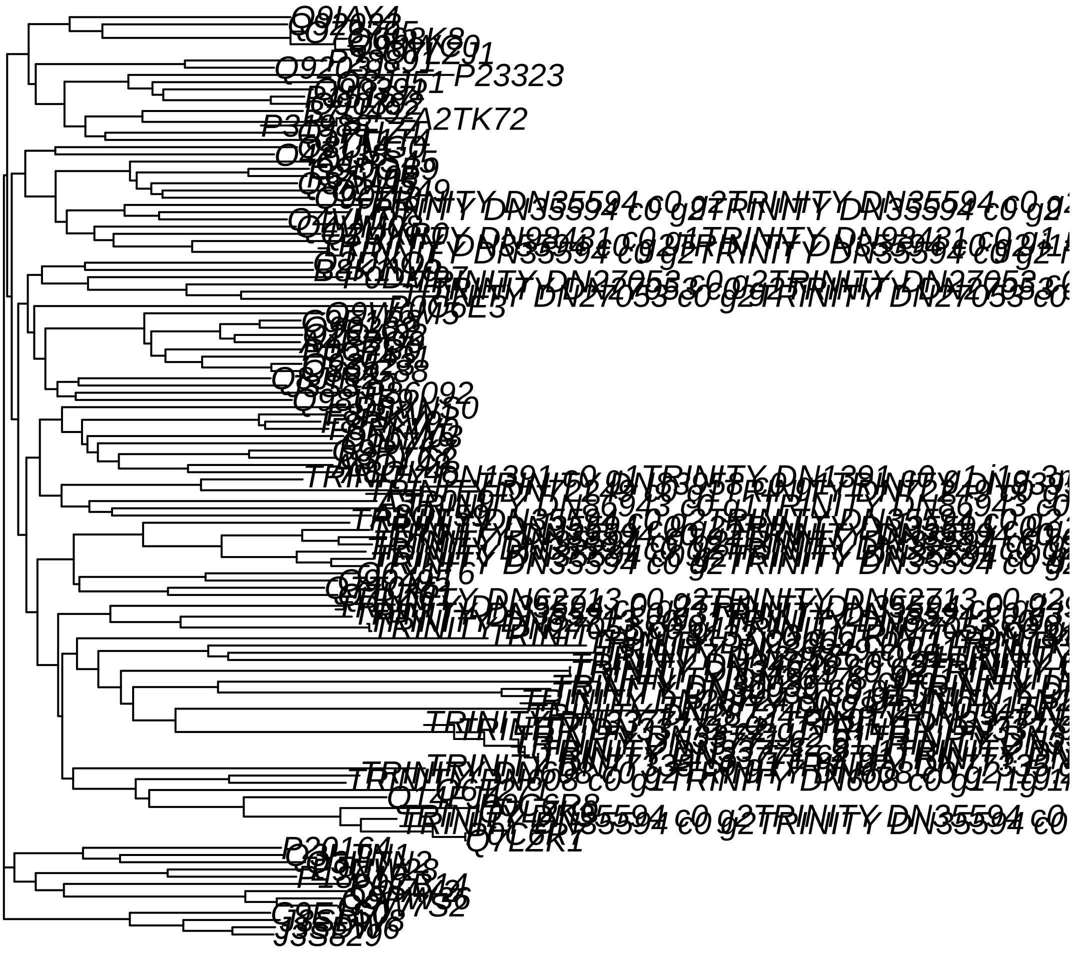

Q9IAY4
P23323
A2TK72
Q7L2R1
P20164
J3SBP6
